# Snake Envenoming: A Disease of Poverty

**DOI:** 10.1371/journal.pntd.0000569

**Published:** 2009-12-22

**Authors:** Robert A. Harrison, Adam Hargreaves, Simon C. Wagstaff, Brian Faragher, David G. Lalloo

**Affiliations:** Liverpool School of Tropical Medicine, Liverpool, United Kingdom; Women's and Children's Hospital, Australia

## Abstract

**Background:**

Most epidemiological and clinical reports on snake envenoming focus on a single country and describe rural communities as being at greatest risk. Reports linking snakebite vulnerability to socioeconomic status are usually limited to anecdotal statements. The few reports with a global perspective have identified the tropical regions of Asia and Africa as suffering the highest levels of snakebite-induced mortality. Our analysis examined the association between globally available data on snakebite-induced mortality and socioeconomic indicators of poverty.

**Methodology/Principal Findings:**

We acquired data on (i) the Human Development Index, (ii) the Per Capita Government Expenditure on Health, (iii) the Percentage Labour Force in Agriculture and (iv) Gross Domestic Product Per Capita from publicly available databases on the 138 countries for which snakebite-induced mortality rates have recently been estimated. The socioeconomic datasets were then plotted against the snakebite-induced mortality estimates (where both datasets were available) and the relationship determined. Each analysis illustrated a strong association between snakebite-induced mortality and poverty.

**Conclusions/Significance:**

This study, the first of its kind, unequivocally demonstrates that snake envenoming is a disease of the poor. The negative association between snakebite deaths and government expenditure on health confirms that the burden of mortality is highest in those countries least able to deal with the considerable financial cost of snakebite.

## Author Summary

Every year snake envenoming kills more people in the tropics than some of the world's recognised neglected tropical diseases (NTDs), including schistosomiasis and leishmaniasis. While lacking the epidemic potential of an infectious/vector-borne disease, snake envenoming in rural tropical communities has as great a medical mortality, if not morbidity, as the NTDs. The recent categorisation of snake envenoming as an NTD is an important advance that hopefully will result in the wider recognition and allocation of resources, particularly since death from snake envenoming is preventable; antivenom is very effective when the appropriate antivenom is correctly administered. Snake envenoming urgently requires international support to instigate the epidemiological, health education, and effective treatment initiatives that proved so potent in addressing the medical burden of NTDs such as leprosy and dracunculosis. All the global estimates of snake envenoming and deaths from snakebite indicate that mortality is highest in the world's tropical countries. Here we examined associations between the globally available data on (i) snakebite-induced mortality and (ii) socioeconomic markers of poverty. Our data unequivocally establishes that snake envenoming is globally associated with poverty, a distinctive characteristic of the neglected tropical diseases.

## Introduction

**Figure 1:**
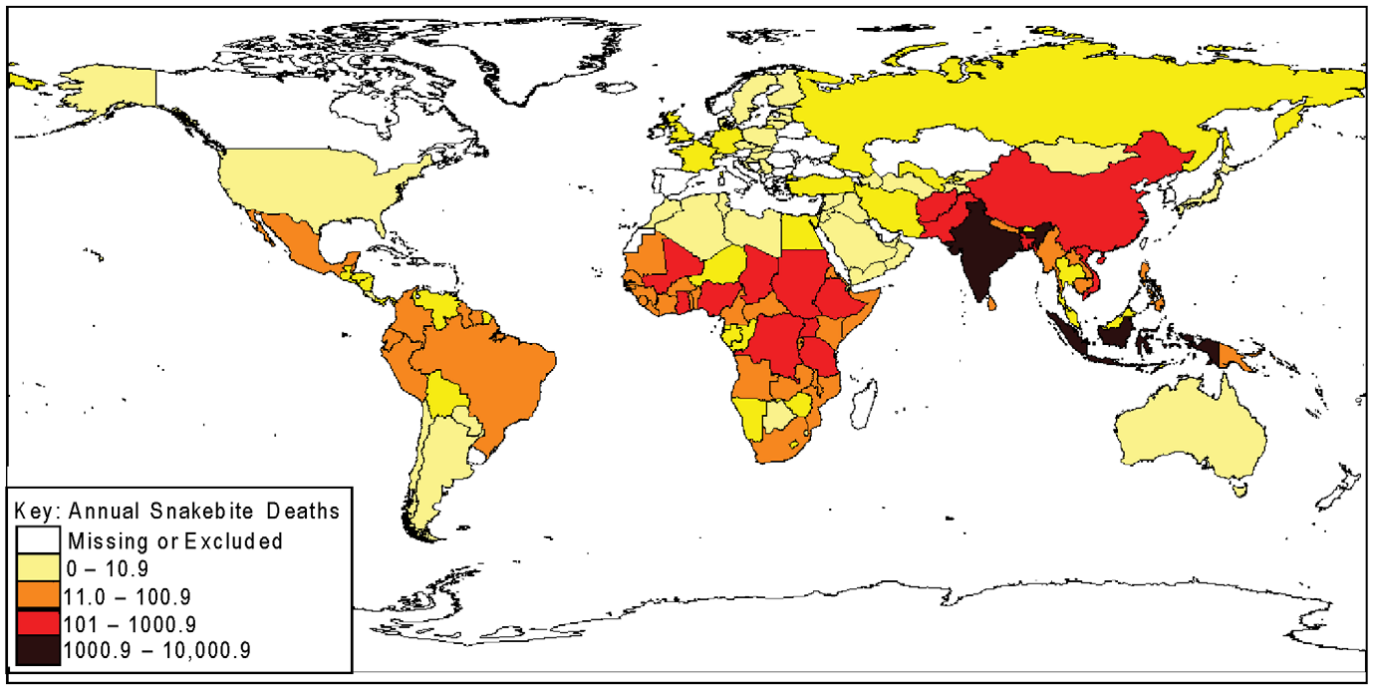
Annual snakebite mortality. Annual estimates of snakebite-induced deaths for 138 countries were obtained from the data published by Kasturiratne et al [4] and depicted on a world map using Epi-info; the darker a country's colour the greater the estimated snakebite mortality – see key for details.

Our knowledge of the global medical burden of snakebite is limited to just a few reports based primarily on either hospital records [1] or the epidemiological literature [2],[3], and more recently, the latter in combination with WHO mortality data [4]. Despite the nearly universal distribution of venomous snakes (the South Pole, Greenland, New Zealand and Madagascar being the major exceptions), each report concludes that the medical importance of snakebite is greatest in the tropics. The vast majority of snakebite-induced deaths (Figure 1) occur in Asia (estimates ranging from 15,400–57,600 deaths pa) and sub-saharan Africa (3,500–32,100 deaths pa) [4]. Populations in this geographic zone also suffer the medical burden of the world's neglected tropical diseases (NTD). Importantly, the number of snakebite-induced deaths doubles the NTD mortality figures for this region due to African trypanosomiasis, cholera, dengue haemorrhagic fever, leishmaniasis, Japanese encephalitis and schistosomiasis [5]–[7]. A major distinctive characteristic of the NTDs is that they are globally associated with poverty [8]. In line with the recent WHO categorisation of snake envenoming as a NTD [9], this analysis was therefore conducted to determine whether the medical burden of snake envenoming is, like the other NTDs, also associated with poverty.

## Methodology

To gain a global perspective of the relationship between poverty and lethal snake envenoming, we entered the now readily available country-specific snakebite mortality data [Supplement 2, [4]] into Epi-Info (version3.5.1) software package [http://www.cdc.gov/epiinfo] to populate the global map of snakebite mortality by country (Figure 1); this differs slightly from the map presented in the report by Kasturiratne et al [4] which presented the same data by geographic region. Entering this country-specific, rather than regional, data provided us with a sufficient spread of data to have confidence in the statistical validity of plotting the mortality data against appropriate socioeconomic indicators. This analysis was performed on the understanding that national estimates of snakebite mortality were not likely to be as accurate as that for the reported wider regions [4], since many of the country estimates were necessarily (because of the lack of available data) extrapolations from neighbouring countries. From amongst the large variety of socioeconomic data available in the public domain, we acquired country-by-country data for ‘Gross Domestic Product Per Capita, US$’ and ‘The Percentage of the Labour Force in Agriculture’ from the CIA World Factbook database [https://www.cia.gov/library/publications/the-world-factbook/]. The data on ‘Per Capita Government Expenditure on Health, US$’ was taken from the World Health Organisation Statistical Information System (WHOSIS) database [http://apps.who.int/whosis/data/Search.jsp]. These three datasets were selected because of the potential link between snakebite mortality and an individual's high-risk agricultural occupation, income and access to healthcare. The Human Development Index (HDI; a composite indicator that reflects life expectancy, education and literacy and standard of living measured by GDP) was also examined and countries categorised into Low (0.1 to 0.499), Medium (0.500 to 0.799) or High (above 0.800) HDI status.

## Results

**Figure 2:**
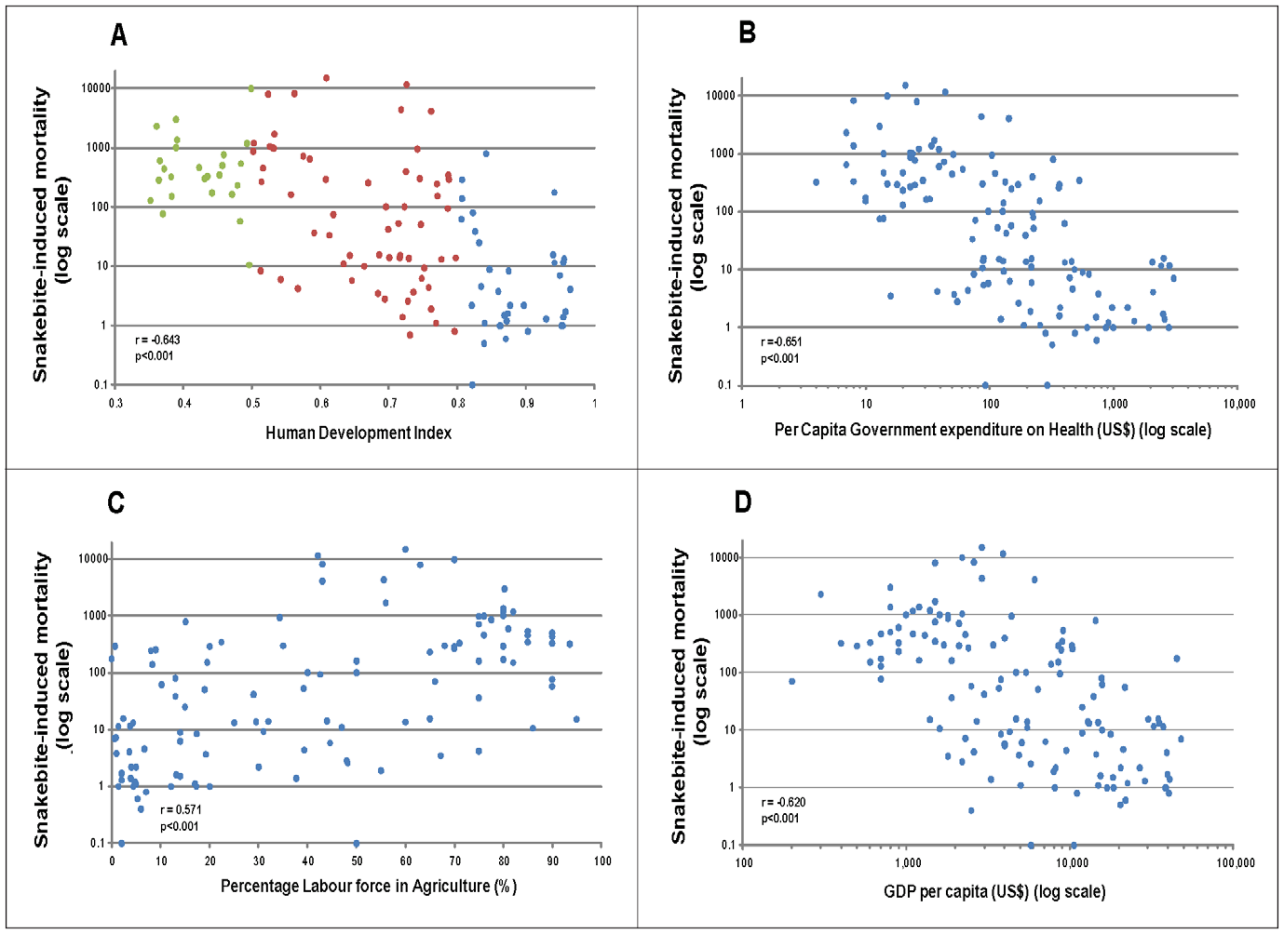
Snakebite mortality and poverty. The annual estimates of snakebite mortality (log scale) for 138 countries were plotted against annual estimates, where available, of **a**) Human Development Index (high - blue dots; medium - red dots; low - green dots), **b**) Per Capita Government Expenditure on Health (US$; log scale), **c**) Percentage Labour Force in Agriculture (%) and **d**) Gross Domestic Product (US$; log scale).

Statistically linear relationships were found between the logarithm of snakebite mortality and the HDI (Figure 2a), the logarithm of Per Capita Government Expenditure on Health (2b), the Percentage of the Labour Force in Agriculture (2c) and the logarithm of GDP Per Capita (2d). The strength of each of these relationships was determined using the Pearson correlation coefficient; all four correlations were numerically strong (range: r = 0.571–0.651) and statistically highly significant (p<0.001).

## Discussion

**Figure 3:**
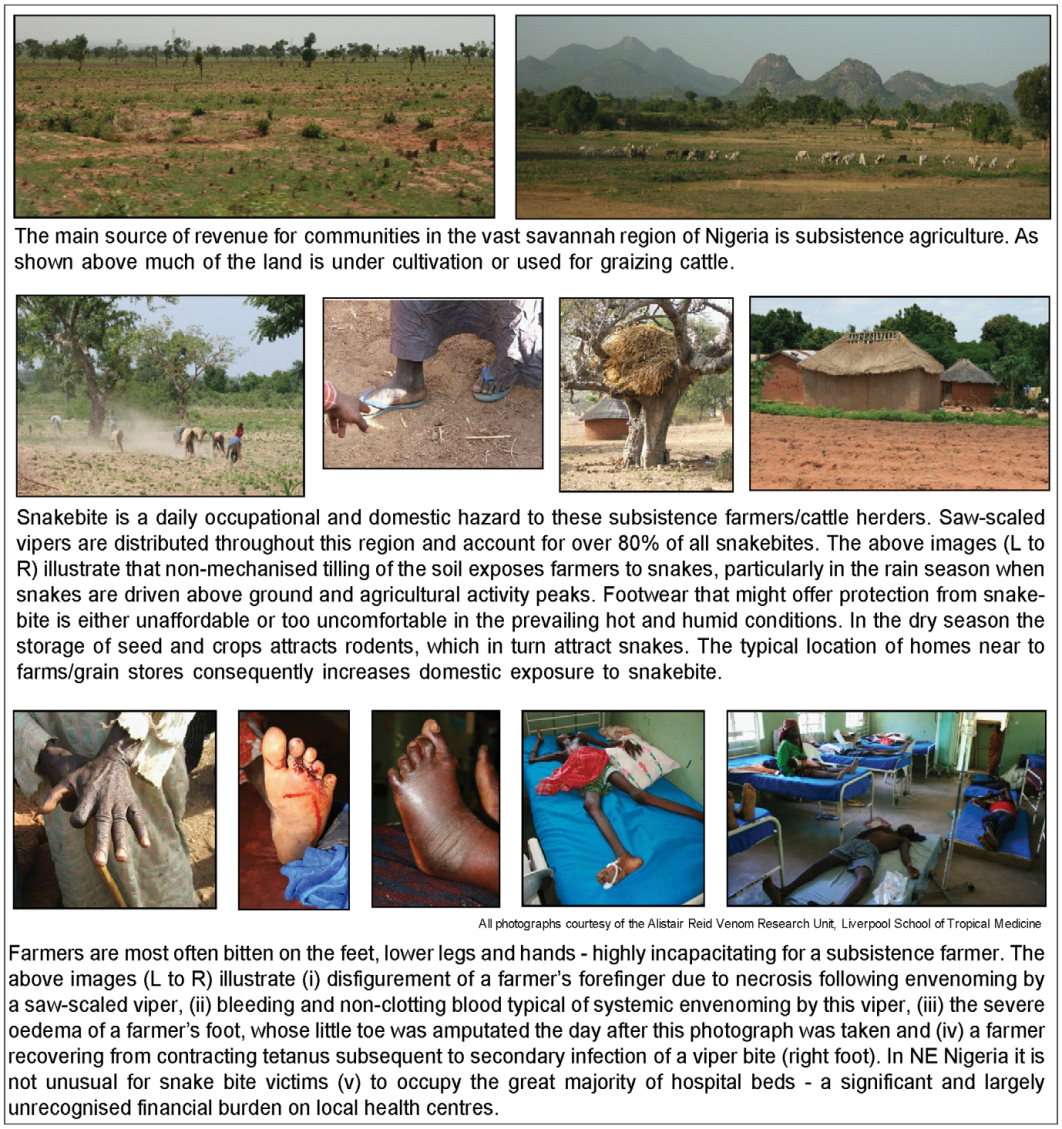
Snakebite and agriculture in rural North-East Nigeria.

The World Bank defined the absolute poverty line as the percentage of a country's population living on an income of less than US$2 per day [10]. Maps depicting countries based on this definition of poverty [11] show a remarkably similar profile as the snakebite mortality map (Figure 1). This relationship between poverty and snakebite mortality is clearly demonstrated by the strong negative correlation between snakebite mortality and both HDI and GDP/capita reported here (Figure 2). The literature on snake envenoming is, like global poverty, rich with references associating rural agriculture with high incidence of disease and death. Taking West Africa as a detailed example, farmers and children in rural communities are consistently identified as being the highest snakebite risk groups in Senegal [12], the Gambia [13], Mali [14], Cote D-Ivoire [15], Ghana [16], Benin [17], Niger [18], Nigeria [19]–[21], Cameroon [22], Gabon [23] and the Congo [24]. The numerous epidemiological reports conducted in Asia and Latin America similarly emphasise that rural subsistent farming communities in these regions also suffer snakebite as a daily occupational hazard (Figure 3). It is not surprising therefore that figures for ‘The Percentage of the Labour Force in Agriculture’ are strongly correlated with global snakebite mortality (Figure 2c). The survival of many of the rural poor is dependent upon their non-mechanised, low-cost farming techniques and it is a cruel irony that it is exactly these practices that place them at such high risk of snakebite, and that their feet, legs and hands are the most frequent anatomical sites of snakebite in Africa [25],[26], Asia [27],[28] and Latin America [29]. The tissue necrotic effects of snake envenoming are thought to afflict many more survivors of snakebite than victims who succumb [30]. Detailed community-based DALY/QALY-type assessments of the true burden imposed by the tissue destructive effects of snake envenoming on these communities are urgently required. There are very few reports in the literature examining the socioeconomic impact of snakebite [31]–[33] and none that we could find concerning the long-term psychological effects of snakebite. It is important that these types of studies are undertaken and the results appropriately disseminated to ensure that governmental, non-governmental and international health agencies understand the medical and sociological consequences of snakebite and the implication for the strategic allocation of scarce health resources.

As with some other NTDs, effective therapy for snake envenoming is available. Antivenom is immunoglobulin purified from the blood of venom-immunised horses and sheep (and rarely other animals). While conceptually simple, the manufacturing process requires GMP-standard production plants and is reliant upon the husbandry and handling of both horses and venomous snakes. Antivenom is therefore relatively expensive (eg, US&$100/vial in S Africa) compared to many other medications used in the tropics. Presumably for this reason, in much of Latin America antivenom manufacture is government-subsidised and antivenom is usually provided free to the patient. This provision and delivery of effective therapy to the at-risk communities may be an important factor in explaining why although snakebite incidence in Latin America is high (129,000), the mortality rates are low (1.78%; 2,300 deaths). Much of Latin America has a high Human Development Index (Figure S1). In contrast, Sub-Saharan African countries, where snakebite victims are charged commercial rates for antivenom (when available), are at the opposite end of the HDI scale. The strong correlation between global ‘Per Capita Government Expenditure on Health’ and snakebite mortality (Figure 2b), illustrates the tragedy that the countries with the highest burden of snakebite mortality are those with the most limited ability to purchase effective antivenom.

There are clearly limitations to this kind of analysis: much of the mortality data is estimated and the indicators, although standard, are still only relatively crude indicators. The limitations are indicated by the anomaly of India, with the highest global snakebite mortality rate but medium Human Development (Supplementary Figure 1) and Human Poverty [11] indices. This is likely to reflect factors such as the huge variation of income within India and might also be explained by complex considerations of population and venomous snake densities, antivenom effectiveness and clinical practices and guidelines [34]. Nevertheless, with only occasional exceptions, our approach is robust in demonstrating the relationship of snakebite to socioeconomic markers of poverty.

The clinical effectiveness of most antivenoms means that snakebite is, in WHO parlance, a ‘tool-ready NTD disease’. This is encouraging: unlike some NTDS, the principles of producing a therapy against snakebite are established, albeit with scope for considerable improvement. As detailed in the WHO Global Plan to Combat NTDs [8], what is required now to resolve the problems of snake bite is a coordinated effort to (i) assess the medical burden (to determine the scale and location of the therapeutic need), (ii) where possible, to integrate snakebite initiatives with those currently pursued for other NTDs, (iii) strengthen health care systems by appropriate capacity building initiatives, (iv) develop communication systems to disseminate ‘disease burden’ information to improve advocacy/public awareness, (v) improve access to affordable, effective treatment and (vi) establish a framework of implementation and evaluation. The scale of this undertaking is daunting but the recent recognition by the WHO that snakebite is a NTD [9] and the establishment of the Global Snakebite Initiative [7] by the International Society of Toxinology is evidence that that the process has already started and that there is enthusiasm for the task.

One of the major hurdles will be to ensure the affordability and effectiveness of antivenom. For most NTDs, once the effectiveness of a treatment has been established, it can be utilised worldwide - which improves commercial economies of scale and encourages widespread pharmaceutical support and drug donation [8]. In contrast, the clinical and geographic effectiveness of antivenom is restricted to the species of snake whose venom was used in its manufacture. This severely limits the implementation of economies of scale and, by inference, the commercial incentives for the involvement of ‘large pharma’. In some regions such as sub-Saharan Africa, until very recently there was only one source of effective antivenom and a crisis in antivenom supply to the continent [35],[36]. Reports of this ‘therapeutic vacuum’ attracted the commercial influx of ineffective antivenoms manufactured from venoms from non-African snakes [37]–[41].

Therefore, despite the effectiveness of current antivenoms, there are compelling reasons to encourage research to broaden the geographic efficacy and improve the commercial viability of antivenom therapy. There are encouraging experimental developments in this area, including ‘antivenomics’ [42] and ‘epitope-string immunogen’ [43],[44] approaches, whose objectives are to maximise the clinical and snake-species efficacy of snakebite serotherapy and minimise manufacturing costs. There are an estimated 20–94,000 snakebite deaths annually, predominantly occurring in the rural poor in the tropics. Improved antivenoms, implementation of the WHO-recommended strategies and above all, international recognition of the importance of the problem could help to reduce many of these deaths.

## Supporting Information

Figure S1 Human Development Index.
(1.02 MB EPS)
